# Intra-Peritoneal Injections of Chloroform to Produce General Anaesthesia

**Published:** 1913-02

**Authors:** 


Intra-Peritoneal Injections of Chloroform to Produce General Anaesthesia.—G. Jeanneney, ibid., September 20, 1912, describes various experiments, animal and human, showing that anaesthesia can be produced with approximately ½ c. c. per kilo of body weight, 10% and later 50% mixtures of chloroform and glycerin and of 50% in sterilized olive oil were used. However, considering the danger of perforating viscera (a cat suffered perforation of the bladder and the liver showed intoxication), degenerative changes, etc., the author concludes that the method is not practical, either surgically or physiologically. (Note—Investigators often fail to realize that a carefully conducted series of experiments with a negative result are usually just as valuable as one leading to positive recommendations. A prize award was refused, a few years ago, because the author, after much expenditure of time and monew, was honest enough to disclaim brilliant results and presented his conclusions with numerous qualifications.)

